# Hospital Meetings, &c.

**Published:** 1900-03-17

**Authors:** 


### HOSPITAL MEETINGS, &C.

### EAST LONDON HOSPITAL FOR CHILDREN.

Mr. H. W. Trinder, the chairman, presided at the thirty-first annual meeting of the East London Hospital for Children and Dispensary for Women, Shadwell, on the 28th ult. In moving the adoption of the report he observed that the Royal Family had assisted them in two ways. The Duke of Connaught took the chair at the festival dinner in May, while the Duchess of York had become a patroness of the institution. During the year they had finally parted with the site of the old hospital, the birthplace of that now great institution. They had done so with some sentimental reluctance; but the money obtained would be applied to the purchase of land which would give them more growing room, and which they found necessary in connection with the newly-built operating theatre and additional ward. This latter had been the chief feature of the year. Their old theatre was built many years ago; the new one, now practically completed, was fitted with the latest and most modern appliances; while the new ward for surgical cases would relieve the pressure on the medical side. Their financial position during the year had been very satisfactory. Their income had increased, though their legacies had decreased, and they had been enabled to invest £1,100 in the building and to advance £300 towards the completion of the convalescent home. This, of course, was not investment in income-producing securities, but it was invested for the benefit of the hospital and of the children who came to them. They had to lament the death of the Rev. Canon Akers, which created a vacancy on the

Board of Management. This had been filled by the election of Mr. Fletcher, of Bognor, who, being on the spot, was able to give attention to the home. No less than 317 cases had been admitted to that home, and it was of very great benefit to patients who had recently undergone operations. They hoped to be able to open a further eight beds at Bognor shortly, bringing the number up to 28. They regretted that the erection of a ward for whooping-cough must for the present stand over; but thanks to the munificence of Mr. Charles Prescott, who had added to his many gifts one of £550 for the purpose, they would shortly have a laundry and sanatorium. The report was adopted and the general committee re-elected, as was also the Board of Management, with the addition of the names of Mr. W. Sims Horner and the Rev. Father Amigo. Mr. Charles Cheston was re-elected treasurer, and the proceedings closed with the customary votes of thanks.

### HOSPITAL FOR CHILDREN AND WOMEN.

The Lord Mayor (Alderman A. J. Newton), the president, who was accompanied by the Lady Mayoress, presided on Friday afternoon, March 2nd, at the eighty-fourth annual meeting of the governors and subscribers of the Royal Hospital for Children and Women, held at the institution, Waterloo Bridge Road.

The annual report stated that, notwithstanding the largely increased amount of good work done during the year, the committee had to report no serious deficiency of funds. Mr. W. H. A. Jacobson, so long surgeon to the hospital, had resigned that position, but had accepted office as consulting surgeon, Mr. Sheild being now surgeon instead of assistant surgeon, Mr. A. D. Fripp being elected to the latter position.

Accompanying the report is issued an appeal for funds for the extension and rebuilding of the hospital, which was originally a City charity—founded in Doctors' Commons in 1816 and removed to its present site in 1823. In their appeal the committee point out that the 52 beds and cots, which have represented the accommodation since 1875, are now inadequate to meet the demands made on the institution, the patients having steadily increased during the past few years, numbering last year 627. The facilities for rebuilding are very great, owing to the reversion to the freehold of six adjoining houses having been purchased in 1881, and the leasehold interests of the buildings in this portion have been mostly acquired. The committee proposes to erect on that site the first half of the new hospital, and on its completion to demolish the present structure and eventually establish a hospital of 200 beds, 100 of which will be immediately thrown open. The estimated cost of the whole is about £25,000. The present assured income, however, is about £2,000 per annum less than the expenditure, but with the increased and improved accommodation the committee looks confidently for greater public support. Various subscriptions have already been promised.

The 627 in-patients in 1899 compares with 599 in 1898, whilst the out-patients have been 9,334, against 8,777 in 1898, the attendances being 37,337, compared with 33,528. The ordinary receipts last year amounted to £3,070, and the extraordinary income to £1,213; the ordinary expenditure being £4,159, and the extraordinary expenses £313, leaving liabilities amounting to £1,936, of which £1,150 is due to the bankers.

The Lord Mayor, in moving the adoption of the report, said he had been unable to give all the attention he desired to the claims of the institution, as at the Mansion House, as they all knew, everybody had been extremely busy during the past few weeks. But he could assure the meeting that it was common repute that this was a glorious hospital, and deserving of all their attention and the support of the public. The time had now arrived when an effort must be made to extend the institution, which was abreast of the times and doing such good work. He moved, as an addition to the resolution, a motion to the effect that the hospital has strong and peculiar claims upon the City of London; that the time has now arrived when it should be extended and rebuilt on modern lines; and that a subscription list be at once opened for that purpose and to free it from debt.

Sir Edwin Durning Lawrence, Bart., M.P., chairman of the committee, seconded, and in doing so outlined the earlier negotiations for the acquisition of the property secured for the purposes of the extension.

The report and the motion dealing with the appeal were unanimously adopted, and the thanks of the meeting having been heartily accorded to the officers and medical staff, a vote of thanks to the Lord Mayor for presiding concluded the proceedings.

### CITY OF LONDON HOSPITAL FOR DISEASES OF THE CHEST, VICTORIA PARK.

The fifty-second annual meeting of the City of London Hospital for Diseases of the Chest, Victoria Park, was held at Gresham College on Monday, 5th inst., Mr. Joseph Benson, L.C.C., chairman of the committee of management, presiding. It will be remembered that at the last annual meeting a very animated discussion was raised by a large number of governors present, representing East-end working men's societies, respecting the inquiry system in force, it being eventually decided that the committee would meet and discuss the question with delegates from the societies at the hospital. Mr. Dudley Ryder, the secretary, now read the minutes of that special meeting, from which it appeared that on March 29th the Committee of Management and about forty representatives of the working men's societies were present, and that the Chairman made an announcement in the following terms: "The committee have decided to withdraw the questions now printed on the back of the out-patients' letters, it being clearly understood that the various working men's societies on their part will undertake to make every possible investigation as to whether candidates applying to them for letters are really eligible for treatment at this hospital." The entry in the minutes continued: "The Chairman wished the members of the East-end societies to clearly grasp that this system of inquiry had been adopted purely as an experiment at the instigation of the constituents of the Hospital Sunday Fund; this period of experiment expired on March 10th. The Chairman further announced that so far as the societies are concerned the Committee of Management had decided to accept letters stamped with the stamp of their society or signature of the secretary of such society, after the name and address of the patient had been filled in, without subjecting the bearer of such letter to the ordeal of being questioned by the inquiry officer; but should it subsequently transpire that any such patient was apparently abusing the hospital whilst in a position to pay for treatment, then the case would be reported to the secretary of such society, and the onus of inquiry into this and all such cases would thus be thrown upon the societies themselves." The report now presented stated that the inquiry system as now conducted was found to work smoothly and with satisfaction to all parties, and the committee had the strongest reasons for believing that the mere presence of the inquiry officer acted as a wholesome deterrent against those who might otherwise endeavour to abuse the privileges of the charity, and the Chairman added that after three months' inquiry they found that not more than 2 per cent of the applicants for relief were not fit subjects for it. Turning to the accounts, he said that financially they found themselves in a very much improved condition. They commenced the year with a balance of £4,140, and closed with £3,952. But during the year they had spent £420

on the erection of balconies on the southern side of the hospital, so that patients might have the benefit of the "open air treatment," and they had also added £2,000 to their investments. The year had also been a satisfactory one as far as the patients and beds were concerned. They now had 130 beds open; two and a-half years ago they had but 40. The number of in-patients for the year was 1,021, an increase of 330, or nearly one-third. The out-patients, on the other hand, were rather less, viz., 15,260, and this they attributed to the presence of the inquiry officer. They were glad of the amicable settlement in this matter which had been come to. They felt that they had their subscribers behind them, and that it was the right thing to do their best to keep away loafers when they were well able to pay for medical attendance. The little difficulty with regard to the working of the arrangement which cropped up at the last meeting had been satisfactorily settled. They had a pleasant conference, and since then everything had gone on very harmoniously and satisfactorily without a sign of friction. Continuing his review of the year, the Chairman said that although they had so many more beds open, their expenses were £1,200 less than in 1896, a reduction due to careful supervision. Their annual subscriptions, amounting to £2,838, again showed an increase of £174. During the past three years this increase amounted to £280 per annum. Their legacies during 1899 were £3,230, as compared with £692 in 1898. Last year the Prince of Wales's Fund had given them a subscription of £1,000 on condition that they increased their beds by 15. This year they had increased it to £1,500 on the further condition of the opening of ten more beds. He thought they could not have stronger evidence that the hospital was well managed than that. Their matron had volunteered for South Africa, and sailed on Saturday, but ample arrangements had been made so that the work should not suffer in her absence. He wished to bear witness, on behalf of the committee, to their appreciation of their secretary. He had been indefatigable in his exertions for the good of the institution, and in stirring up interest in every direction. In conclusion, he was sure all would be glad to learn that since the report had been issued an anonymous friend had given £1,000 for the endowment of a bed in memory of a friend—an example he should like to see followed by many others.

The report and accounts were agreed to without discussion. Sir Edward Sassoon was re-elected treasurer, and the customary votes of thanks brought the proceedings to an end.

### WEST LONDON HOSPITAL.

The annual meeting of the governors of the West London Hospital in Hammersmith Road was held on Monday, March 5th, in one of the unoccupied wards of the new extension of the building, Lord Glenesk presiding.

The report of the Board of Management, which was read by the Secretary, dealt in detail with the improvements which had been carried out during the past year, and also with the special efforts made to provide additional income. The board specially called attention to the fact that mainly owing to the unusually small amount of £310 received as legacies, and that no donation of a large amount had been given, the total income on the maintenance and building accounts amounted to only £8,096, compared with £10,047 in 1898. At the same time, owing to the getting into use of two of the new wards and the construction of the new out-patients' consulting rooms and board room, the total expenditure of the two accounts amounted to £11,571 against £9,932. The special thanks of the board were accorded to a number of local business firms and organisations who had sent substantial sums to the hospital, whilst the board had received from the Prince of Wales's Fund £1,000, as compared with £500 in 1898; and from the Metropolitan Hospital Sunday Fund £625. The net amount of the debt weighing on the institution somewhat exceeded £12,900. The registrar's report showed that the in-patients under treatment in 1899 were 2,042, against 1,606 in 1898, the number of beds available being 153, against 101; and the total cost of in-patients £7,569, against £5,680. The out-patients numbered 25,191, as compared with 31,170 in 1898, and the total cost of the same was £1,170, compared with £1,241.

The total ordinary income was £6,843, and the extraordinary income £310, whilst the ordinary expenditure was £8,740, compared with £6,922 in 1898, and the total extraordinary expenditure £149. The building account showed an income of £943, and the expenditure under that head amounted to £2,682, an excess expenditure of £1,739.

In moving the adoption of the report, Lord Glenesk said in view of the war being most in their minds at present, it was pleasing to note that the West London Hospital was well represented at the front, for not only had their officer, Mr. G. L. Cheatle, been appointed as a consulting surgeon to the force, but six other members of the hospital staff were now serving in South Africa. The hospitals at home, with their schools and scientific methods of curing diseases and alleviating pain, deserved the cordial support of the public, and he thought such institutions would obtain that support. Further, he did not think that the large sums which were being subscribed for the benefit of our soldiers and their families, and for the relief of the sufferers by the Indian famine, would have the effect of depriving any worthy cause at home of one penny which it might otherwise hope to receive. He knew from personal knowledge that the tendency was all the other way, and in times like the present, when people's hearts were open, their purses were also open. With reference to that particular institution, it was deserving of the warmest support on the ground that it was situated at one extreme of London and surrounded by a large and increasing population. It has been very much improved by the new extensions. He had always held that such institutions should depend for support on the local residents, and it was very satisfactory to know that the West London Hospital had received handsome donations from employers of labour and others residing in West Kensington. He would suggest, as a means of raising funds, the holding of a bazaar, which would be very popular and enlist the support of ladies of the western district, and probably also of Royalty. It was unnecessary for him to dwell on the merits of the hospital; they were well-known to all those present. They had confidence in the governors, and knew that the institution was administered by them most efficiently and with the greatest economy, whilst nothing was left undone to properly provide for the patients.

Colonel Napier seconded the motion, and the report was unanimously adopted. The Bishop of London (Dr. Mandell Creighton) was elected a vice-president, and Lord Rothschild re-elected as treasurer. The retiring members of the Board of Management (with the addition of Rear-Admiral Hand) were re-elected.

In replying to a vote of thanks for presiding, Lord Glenesk offered ten equipped beds for the unoccupied ward in which the meeting took place, and stated he wished brass plates to be put up over them, with the names of Generals Roberts, Kitchener, and Buller, Dr. Cheatle, and the six surgeons of the staff of the hospital who had been mentioned as now serving at the front in the war. The announcement was received with great enthusiasm.

### CHELSEA HOSPITAL FOR WOMEN.

The twenty-ninth annual general meeting of the governors of the Chelsea Hospital for Women was held at the hospital on the 6th inst.

Lord Glenesk presided, and moved the adoption of the report and financial statement. Both were taken as read.

In commenting on the report, the Chairman congratulated the meeting upon the successful issue of a very anxious year. There had been a great deal to undertake, he said, for it was necessary to use every possible precaution to ensure the success of the difficult and critical operations performed in the hospital. The authorities might, however, now safely boast that everything was in perfect order. They possessed a large and convenient operating theatre, the best instruments, and the greatest medical skill. Extraordinary results had been achieved. By means of a delicate recording instrument it had been shown that not only was the patient insensible to pain by the anæsthetic administered, but that the temperature and former physical movements remained practically unchanged, thus proving that she was completely free from the usual shock and agitation occasioned by the operation. A recent paper in the *Lancet* had quoted the Chelsea Hospital for Women as having the lowest death-rate. It was then 2 per cent., and now it had been further reduced to 1½ per cent. The structural improvement of the theatre, and the consequent rearrangement of the wards, the installation of the lift, the electric light, and the hot-water supply had been costly. They had had to face an outlay of £3,000, and had appealed to the public confidently for support. Many had given handsomely; many had collected assiduously; and one friend, who wished to remain nameless, had given two donations of £500 each. This money had been devoted to the improvement of the operating theatre where a mural tablet recording the gift had been placed. Her generosity would vastly mitigate suffering—would facilitate the advance of science along the path of progress. The Chairman then drew attention to the gratuitous and valuable services of the architect, Mr. Emerson, in recognition of which he had been elected a member of the council and an hon. life governor. In dealing with the question of finance, Lord Glenesk remarked that more help, more money, especially more annual subscriptions, were needed. They had lost no fewer than fourteen old friends by death this year, and he earnestly solicited the support of new subscribers. The grant from the Hospital Sunday Fund, the largest ever received, was £356. That from the Prince of Wales's Hospital Fund was less than the last by £50. On the other hand, this Fund had given £50 to the convalescent home. The income from annual subscribers was £1,624, against £1,638 in the previous twelve months. The chief donations had been received from H. M. E., of £1,000; from Mrs. Sington (collected), of £225; from the Baroness de Hirsch, of £200; from Mr. Passmore Edwards, of £105, &c.

Dr. Duncan moved the election of the Council for the year. The names of three new members—Mr. Emerson, Mr. F. J. Dean, and Major-General Henry Wemyss, C.B.—were added to the original number. The resolution was unanimously carried.

Mr. R. J. Beadon put a vote of thanks to the honorary medical staff and other honorary officers in most complimentary terms, and the motion was cordially supported and carried.

### LONDON HOMŒOPATHIC HOSPITAL.

The annual general meeting of the governors, donors, and subscribers of the London Homœopathic Hospital was held on Thursday, March 8th, in the board-room of the new building, Great Ormond Street, Bloomsbury, Earl Cawdor, the treasurer, presiding.

The Secretary (Mr. G. A. Cross) read the fiftieth annual report, which showed that in 1899 the in-patients and out-patients had again exceeded in number those of any previous year, the former having been 1,128, and the latter 20,678, of which 9,883 were renewals. The year's total ordinary expenditure has been £10,126, against an ordinary income of £7,016, leaving a deficit of £3,099. The extraordinary income (legacies, &c.) was, however, £21,076, and the extraordinary expenditure (apart from investments) £93. The deficit on the ordinary account for the four years ended 1898 of £9,556 had been temporarily met by loans from the bankers of £3,000, and £6,556 withheld or withdrawn from the capital account under resolutions of a special meeting held in 1896 authorising a withdrawal of £8,000 on the understanding that the Board of Management should, if practicable, replace the amount. This deficit had been reduced to the extent of £7,894 by the donations secured at the festival dinner on June 21st. The board, however, point out in their report that the amount actually borrowed from the invested funds was £9,556, and that they began the year 1899 with a draft on capital beyond the sum voted of £1,556. The deficit in 1899 being £3,099, the total drawn from capital in December last was £4,656, and the board applied to the meeting for a fresh draft on capital to cover that amount, and in doing so pointed out that such drafts must take place till the income equals the expenditure. In addition to paying off the loan to the bank the board had placed £3,000 on deposit, and had added to the amount of preference shares in the Morgan Crucible Company (already held under a legacy) by taking up an allotment of new preference shares at a cost of £499. The hospital had received awards of £200 from the Prince of Wales's Fund, £625 from the Metropolitan Sunday Fund, and £147 from the Saturday Fund; whilst the legacies received during the year included £12,328 from Dr. J. Say Clarke, and £500 from Miss Jane Mary Barclay.

The annual report of the Convalescent Home at Eastbourne showed a total of 194 residents during 1899, which included 16 nurses of the hospital. The receipts from private nursing had again shown an increase.

Earl Cawdor, in moving the adoption of the report, pointed out that the expenditure had now reached its maximum, and he trusted they might succeed in their endeavour to increase the income so as to balance their expenses.

Mr. J. B. Stilwell (chairman of the board) seconded the motion, and after some comments by Colonel Clifton Brown the report was adopted.

On the motion of Mr. Sidney Gedge, M.P., Law 30 was agreed to be altered by omitting the words "all donations or bequests of fifty guineas and upwards," and inserting instead "all bequests of one hundred guineas and upwards." This alteration will allow of sums of less than £105 being applied to ordinary expenditure, instead of being necessarily invested as hitherto.

The following resolutions were then adopted, on the motion of Captain Cundy: "That this general meeting of the governors, donors, and subscribers of the London Homœopathic Hospital, having in view the continued inadequacy of the annual income to meet the annual expenditure of the hospital, hereby empowers and directs the Board of Management and trustees as follows: (1) That for each of the years 1899, 1900, 1901, and 1902 sums not exceeding £3,000 in each of those years be withheld or withdrawn from the reserve fund and be expended in the discharge of current expenditure; (2) that such sums be refunded to the reserve fund, if and when practicable in the judgment of the Board of Management; (3) that in the event of the available receipts of those years being found, in the judgment of the Board of Management, inadequate to the refunding of such sums after payment of the current expenditure of those years, then such sums as cannot be refunded shall, under the authority of this resolution, and without further condition as to refunding, remain finally appropriated for the use and service of the hospital for the discharge of the current expenditure of those years, and all responsibility of the Board of Management and trustees as to the refunding of such sums shall be hereby discharged and annulled." Sanction was also given to the board as shareholders to take up an advantageous allotment of Morgan Crucible Company's shares; and the proceedings closed with votes of thanks to the officers and staff, and to the chairman for presiding.

### NATIONAL HOSPITAL FOR DISEASES OF THE HEART.

The Earl of Verulam presided at the annual meeting of the National Hospital for Diseases of the Heart and Paralysis on the 8th inst. The report presented stated that the income during the past year, legacies excepted, was £1,995, and the expenditure £2,448, as compared with £2,255 and £2,351 respectively in 1898. One legacy of £240 was received. The deficiency between revenue and expenditure was met by the transfer of £200 from the building fund. The committee expressed regret that owing to the protracted negotiations with the trustees of the Glossop Estate they were unable to carry out the building improvements last summer, but hoped to be able to do so during the current year. Notwithstanding that admissions were suspended for six weeks owing to an outbreak of influenza in the wards, 179 in-patients were admitted, as against 178 in 1898; while the out-patients' attendances numbered 13,020, as compared with 11,404. In consequence of the increase in the number of heart cases, and in accordance with the wishes of the medical staff, the committee had decided to recommend that the treatment of paralysis be discontinued, and that the word paralysis be no longer used in the designation of the institution. The report and accounts having been adopted, resolutions were passed sanctioning the proposed change in the rules and title of the hospital, the committee of management were re-elected, and votes of thanks to them, to the staff, and to Lord Verulam for his services, brought the meeting to a close.

